# When drought helps herbivores before it harms them

**DOI:** 10.1093/jxb/erag265

**Published:** 2026-09-01

**Authors:** Pablo Urbaneja-Bernat, Sara I Zandalinas

**Affiliations:** Plant Immunity and Biochemistry Group, Biology, Biochemistry and Natural Sciences Department, Universitat Jaume I, Castellón, Spain; Department of Biology, Biochemistry and Natural Sciences, Universitat Jaume I, Av. de Vicent Sos Baynat, s/n, Castellón de la Plana, 12071, Spain

**Keywords:** Drought, herbivory, metabolomics, *Pegomya cunicularia*, plant–insect interactions, stress combination, sugar beet, volatile organic compounds

## Abstract

This article comments on:

**Rahman S, Surovy MZ, Vosteen I, Rostás M.** 2026. Metabolic trade-offs in sugar beet under drought and beet leaf miner infestation: implications for herbivore success. Journal of Experimental Botany **77**, 5267–5283. https://doi.org/10.1093/jxb/erag196

This article comments on:

**Rahman S, Surovy MZ, Vosteen I, Rostás M.** 2026. Metabolic trade-offs in sugar beet under drought and beet leaf miner infestation: implications for herbivore success. Journal of Experimental Botany **77**, 5267–5283. https://doi.org/10.1093/jxb/erag196

**Plants rarely experience drought or herbivory attack as isolated stresses, yet their combined effects are still often inferred from single-stress paradigms. In this study, Rahman *et al*. (2026) show that the outcome of drought–herbivory interactions in sugar beet strongly depends on stress intensity. By integrating plant physiology, metabolomics, volatile emissions, and insect performance, they reveal a striking trade-off: moderate drought appears to enhance host suitability for larval performance, whereas severe drought is associated with reduced host attractiveness and lower larval success, probably through effects on both post-oviposition host suitability and volatile-mediated host detectability. Their study highlights why not all droughts are biologically equivalent.**

## Plants under multiple stresses

Understanding how biological systems respond to stress remains a central challenge in biology. Traditionally, this question has been approached through a reductionist framework in which individual stress factors are examined separately. However, in both natural ecosystems and agricultural settings, plants are rarely exposed to a single stress in isolation. Instead, they frequently encounter multiple environmental and biotic challenges that may occur simultaneously or sequentially. This complexity raises an important question: to what extent do conclusions from single-stress experiments capture plant responses under more realistic conditions (Mittler, 2006; Zandalinas *et al*., 2021a). Climate change is expected to further complicate these interactions by reshaping the distribution, intensity, and timing of both abiotic and biotic stress factors. At the same time, it is altering the prevalence, distribution, and impact of biological antagonists across both natural and agricultural systems (Hamann *et al*., 2021; Garrett *et al*., 2022; Leisner *et al*., 2023; Xiong *et al*., 2026). As a consequence, plants are increasingly likely to experience combined stress scenarios in which the outcome cannot be inferred from either factor alone.

Plants have evolved complex defense and acclimation mechanisms to withstand adverse environments, and previous studies have shown that simultaneous or sequential exposure to different abiotic and biotic stresses often triggers molecular and metabolic responses that differ markedly from those induced by each stress individually (Atkinson and Urwin, 2012; Prasch and Sonnewald, 2015; Zandalinas *et al*., 2021b; Pardo-Hernández *et al*., 2024; Pascual *et al*., 2025). Among abiotic stresses, drought is one of the most important constraints on crop productivity worldwide, and both its frequency and severity are projected to increase under future climate scenarios (IPCC, 2023). Drought can strongly influence plant–insect interactions, affecting herbivore feeding, development, and host selection behavior, yet these outcomes are highly context dependent and often vary with drought intensity and duration (Lin *et al*., 2023).

**Fig. 1.**
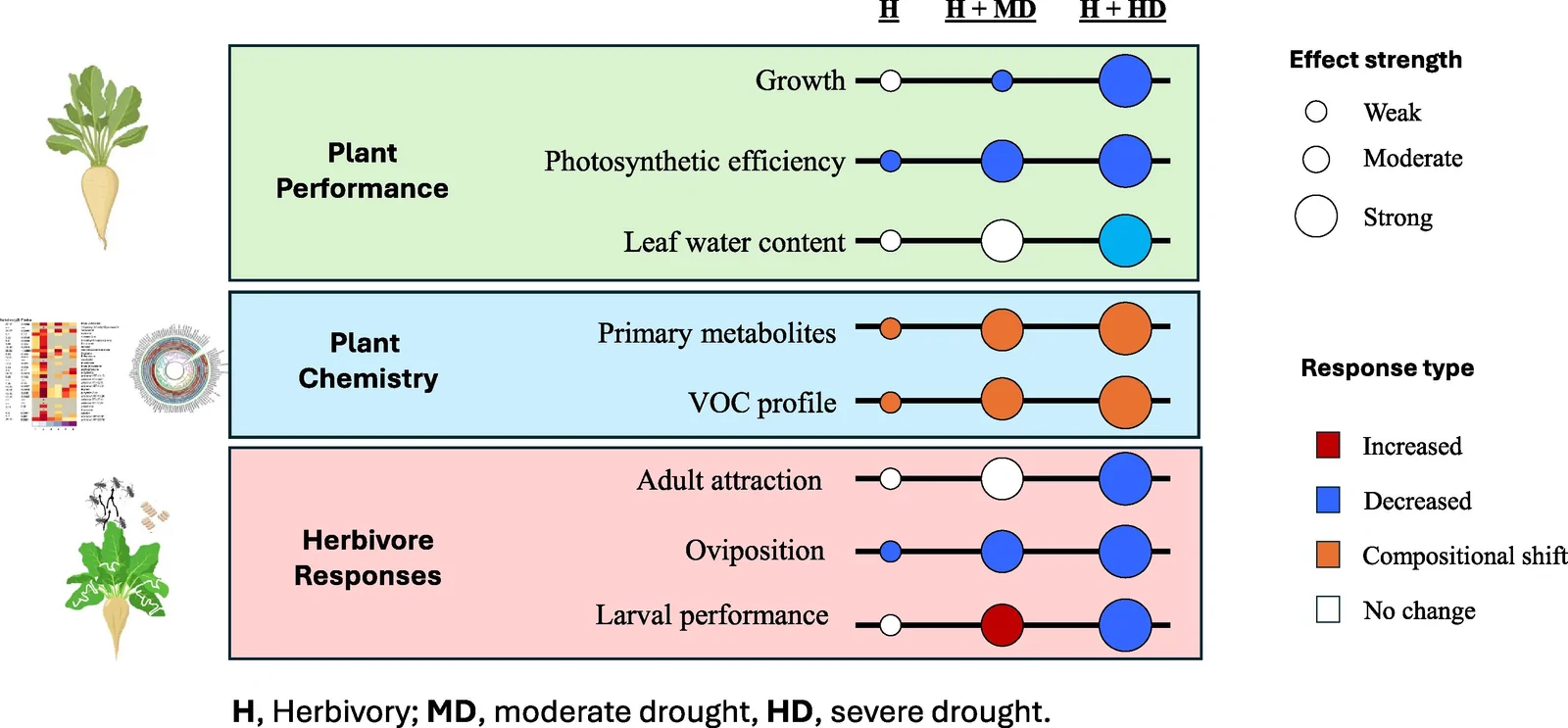
Conceptual summary of the stress-intensity-dependent effects of herbivory and drought on sugar beet performance, chemistry, and beet leaf miner responses. Herbivory (H) alone mainly reduces photosynthetic efficiency and alters plant chemistry. Under moderate drought plus herbivory (H + MD), plant growth declines, leaf water content remains largely unchanged, plant chemistry is reconfigured, oviposition decreases, and larval performance improves. Under severe drought plus herbivory (H + HD), plant performance is strongly reduced, plant chemistry is extensively altered, adult attraction and oviposition decline, and larval performance is impaired. Circle size indicates effect strength, and colors indicate response type.

Rahman *et al*. (2026) provide a refined example of this complexity. Using sugar beet and the beet leaf miner *Pegomya cunicularia* (Diptera: Anthomyiidae), they show that the outcome of drought–herbivory interactions strongly depends on stress intensity. By combining plant physiology, metabolomics, volatile organic compounds (VOCs), insect behavior, oviposition preference, and developmental performance, the authors reveal that drought appears to reshape two key dimensions of host suitability at the same time: the inferred nutritional suitability of plant tissues and the detectability of the host through volatile cues (Fig. 1). This dual perspective is what makes the study notably insightful.

## Drought intensity reshapes host suitability and detectability

One of the clearest outcomes of the study is that drought does not affect herbivore success in a linear way. Under moderate water limitation, sugar beet leaves appear to become a more suitable substrate for development of the beet leaf miner larval, based on metabolite profiles and insect performance. Combined moderate drought and herbivory increased the abundance of central metabolites, particularly amino acids and organic acids, while the sugar to amino acid ratio declined. Several stress-associated amino acids (e.g. glycine, leucine, isoleucine, valine, proline, alanine, asparagine, threonine, and tyrosine) accumulated under this combination of abiotic and biotic stress, suggesting that moderate drought may be associated with an enrichment of host tissues in compounds potentially relevant for herbivore growth. The herbivore experiments supported this interpretation, with moderately drought-stressed plants showing greater feeding damage, higher hatching rates, and larger pupae and adults of the beet leaf miner, although this improved larval performance did not translate into increased female oviposition preference. In ecological terms, this is a key result: moderate stress may enhance post-oviposition host suitability before water limitation becomes physiologically restrictive. The plant is stressed enough to alter metabolism but not yet stressed enough to become a poor feeding substrate for developing larvae. This helps explain why the relationship between drought and herbivore success is often inconsistent across studies (Lin *et al*., 2023). Drought does not operate as a simple on/off factor. Instead, moderate stress may open a window in which the host plant becomes temporarily more profitable for larval development (Gely *et al*., 2020), because tissue hydration remains sufficient, while metabolism shifts towards a profile consistent with enhanced host suitability. In this case, moderate drought appears to generate precisely such a window for the larval performance of *P. cunicularia,* rather than adult host preference.

The ecological picture changes considerably when drought becomes severe. High drought reduced plant growth, leaf area, and biomass, and, importantly, significantly decreased leaf water content. This marks a crucial difference from moderate drought, under which tissues remained exploitable despite metabolic reprogramming, whereas under severe drought the host crossed a physiological threshold that made it less suitable for herbivore development. Larval performance declined, and feeding damage was reduced compared with the moderate drought treatment. Yet the most compelling contribution of the study is that severe drought appears to affect not only host suitability but also volatile-mediated host detectability. VOC emissions were altered both qualitatively and quantitatively across treatments. Total VOC emission per plant decreased with increasing drought, and the volatile blend itself was restructured, including the disappearance of some compounds from drought-stressed plants and the loss of sesquiterpenes in highly stressed, leaf miner-infested plants. Thus, severe drought is likely to change not only the host suitability, but also how readily the host can be located by the herbivore. Female beet leaf miners were more attracted to control plants than to highly drought-stressed plants, and oviposition strongly declined with drought severity (Fig. 1). This offers an important mechanistic insight, as herbivore success depends on both host location and subsequent larval performance. Severe drought, therefore, does not simply create a less suitable host for larval development; it is also associated with reduced attractiveness to ovipositing females.

## Broader implications for ecology and pest management

The implications of this work extend beyond sugar beet and a single leaf miner. As drought episodes become more frequent and unpredictable (IPCC, 2023), crop systems will increasingly experience combined abiotic and biotic stress scenarios in which the intensity of water deficit can change the direction of plant–insect interactions. This is important for prediction. Pest outbreaks under climate change are often discussed in broad terms, in general through extreme climatic scenarios defined mainly by temperature and relative humidity (Urbaneja-Bernat *et al*., 2019), but Rahman *et al*. (2026) show that different components of herbivore performance and behavior may respond positively or negatively depending on the level of drought stress to which the plant is exposed.

These findings also have important implications for crop protection strategies. If moderate drought can increase larval performance by altering tissue metabolite profiles in ways consistent with enhanced host suitability, then crops may become vulnerable before symptoms of severe stress are visually obvious. Conversely, severe drought may reduce oviposition and larval success, but only at a substantial cost to plant growth and productivity. Thus, the relationship between drought and pest pressure is unlikely to be translated into simple management rules. Instead, a more realistic framework should integrate plant water status, metabolite profiles associated with host suitability, and volatile-mediated insect behavior.

A further step will be to move beyond dual stress and towards more realistic multifactorial scenarios. In the field, drought often co-occurs with elevated temperature, fluctuating humidity, nutrient limitation, or attack by multiple organisms. The results revealed here suggest that such combinations will not produce uniform responses but will reposition plants within a multidimensional space defined by nutritional suitability, detectability, and defense. Although exploring the full multifactorial combination of abiotic and biotic drivers would be the ideal next step, doing so remains a highly challenging task. This complexity should be addressed with particular caution and will probably require coordinated efforts from large, interdisciplinary teams capable of considering the many interacting factors involved. Identifying the relevant thresholds and key parameters within that space may become one of the key challenges for future research on plant–insect interactions.

At the same time, this study opens up several questions. Rahman *et al*. (2026) focus mainly on central metabolism and volatile emissions, but future work could test whether drought intensity similarly reshapes specialized metabolites and direct defense traits. It would also be highly useful to determine how natural enemies respond to the same drought-induced changes in plant odors. If severe drought is associated with reduced host attraction or detectability for herbivores, does it also affect the foraging efficiency of parasitoids and predators? Alternatively, might different trophic levels respond in different ways to the same volatile changes? These are particularly relevant questions for integrated pest management, where drought may alter not only pest pressure but also the reliability of biological control.
